# The Impact of Social Media on Adolescent Body Image: A Comprehensive Review

**DOI:** 10.1192/j.eurpsy.2025.1124

**Published:** 2025-08-26

**Authors:** M. Demetriou, V. Anagnostopoulou, V. Markatis, M. Peyioti, P. Argitis

**Affiliations:** 1 Psychiatry Department, General Hospital of Corfu, Corfu; 2 University General Hospital of Alexandroupolis, Alexandroupolis, Greece

## Abstract

**Introduction:**

Social media has become a powerful influence on adolescent body image. Platforms like Instagram and TikTok, which focus on appearance, often promote idealized body standards, leading young users to internalize unrealistic beauty ideals. This has resulted in increasing body dissatisfaction and negative mental health outcomes, as adolescents seek validation through likes and comments.

**Objectives:**

This review examines how social media exposure affects body image and emotional wellbeing in adolescents, particularly whether it contributes to negative outcomes like low self-esteem, body dissatisfaction, and mental health issues.

**Methods:**

A review of international studies published in the last five years was conducted using PubMed and Google Scholar. Search terms included “social media,” “body image dissatisfaction,” “mental health,” and “adolescents.” A total of 26 studies that met the criteria were analyzed.

**Keywords:**

social media, body image dissatisfaction, adolescents, mental health, eating disorders, self-esteem, COVID-19

**Results:**

The studies consistently revealed a strong link between frequent social media use and negative body image. Both male and female adolescents reported increased body dissatisfaction, though most studies focused on females. Social media exposure was also linked to higher risks of eating disorders and a drive for thinness.

Frequent users of appearance-focused platforms experienced reduced self-esteem and heightened levels of anxiety and depression, with social comparison behavior worsening these effects. Adolescents’ body image was further influenced by peer and parental validation, with peer approval playing a critical role in shaping their self-perception. The negative impacts of social media were particularly exacerbated during the COVID-19 lockdown due to increased isolation.

**Conclusions:**

Social media platforms centered on appearance have a significant negative impact on adolescents’ body image and mental health. These findings highlight the need for interventions promoting media literacy, critical social media engagement, and support from parents and educators to mitigate these effects.

**Disclosure of Interest:**

None Declared

